# Where We Live Matters: Residential Influences on Health and Well-Being

**DOI:** 10.1093/geroni/igaa057.2242

**Published:** 2020-12-16

**Authors:** Yee To Ng, Elizabeth Munoz, Markus Schafer

## Abstract

Growing evidence indicates that residential contexts are implicated in the health and well-being of older adults. Operationalization of these contexts varies and includes psychosocial, physical and socioeconomic neighborhoods, and more proximal contexts (e.g., home environment). We aim to bring together a diverse set of papers focused on the living environment to understand how contextual factors are associated with individual outcomes. Muñoz and colleagues applied a lifespan perspective by evaluating associations between current and childhood neighborhood perceptions on cognitive health. Their results indicated that the association between perceived neighborhoods and cognition in adulthood was moderated by childhood neighborhoods. García and Ailshire contextualized the types of neighborhoods in which older Latinos live and how these influenced diabetes risk. They identified neighborhood clusters characterized by racial/ethnic and socioeconomic compositions and found that predominantly Latino neighborhoods with low SES were more likely to have diabetes compared to other neighborhood clusters. This symposium will also focus on the more proximal environment. Lee and Ailshire examined the neighborhood and home environment and found that the home’s proximity to green space and level of clutter within the home was associated with increased fall risks in older adults. Fingerman and colleagues coded older adults’ living spaces and found that personality type was associated with room conditions. Altogether, the presentations highlight the relevance of context measured across multiple levels of analyses and dimensions of well-being outcomes in aging individuals. Dr. Markus Schafer will provide a discussion of these findings and address the challenges and opportunities for future research.

